# Indirect Observation in Everyday Contexts: Concepts and Methodological Guidelines within a Mixed Methods Framework

**DOI:** 10.3389/fpsyg.2018.00013

**Published:** 2018-01-30

**Authors:** M. Teresa Anguera, Mariona Portell, Salvador Chacón-Moscoso, Susana Sanduvete-Chaves

**Affiliations:** ^1^Faculty of Psychology, Institute of Neurosciences, University of Barcelona, Barcelona, Spain; ^2^Faculty of Psychology, Department of Psychobiology and Methodology of Health Sciences, Universitat Autònoma de Barcelona, Barcelona, Spain; ^3^Facultad de Psicología, Universidad de Sevilla, Seville, Spain; ^4^Departamento de Psicología, Universidad Autónoma de Chile, Santiago, Chile

**Keywords:** indirect observation, mixed methods, textual materials, verbal behavior, systematic observation, quantitizing

## Abstract

Indirect observation is a recent concept in systematic observation. It largely involves analyzing textual material generated either indirectly from transcriptions of audio recordings of verbal behavior in natural settings (e.g., conversation, group discussions) or directly from narratives (e.g., letters of complaint, tweets, forum posts). It may also feature seemingly unobtrusive objects that can provide relevant insights into daily routines. All these materials constitute an extremely rich source of information for studying everyday life, and they are continuously growing with the burgeoning of new technologies for data recording, dissemination, and storage. Narratives are an excellent vehicle for studying everyday life, and quantitization is proposed as a means of integrating qualitative and quantitative elements. However, this analysis requires a structured system that enables researchers to analyze varying forms and sources of information objectively. In this paper, we present a methodological framework detailing the steps and decisions required to quantitatively analyze a set of data that was originally qualitative. We provide guidelines on study dimensions, text segmentation criteria, *ad hoc* observation instruments, data quality controls, and coding and preparation of text for quantitative analysis. The quality control stage is essential to ensure that the code matrices generated from the qualitative data are reliable. We provide examples of how an indirect observation study can produce data for quantitative analysis and also describe the different software tools available for the various stages of the process. The proposed method is framed within a specific mixed methods approach that involves collecting qualitative data and subsequently transforming these into matrices of codes (not frequencies) for quantitative analysis to detect underlying structures and behavioral patterns. The data collection and quality control procedures fully meet the requirement of flexibility and provide new perspectives on data integration in the study of biopsychosocial aspects in everyday contexts.

## Introduction

Psychological science has shown a growing interest in the study of everyday life. New methodologies have been proposed for the within-person study of real-time biopsychosocial aspects in their natural settings (Bolger et al., 2003; Conner and Lehman, 2013; Reis, 2013; Portell et al., 2015b,c). New technologies have made it possible to record spontaneous behavior—that is, behavior that is not elicited by a researcher but forms part of the subject's behavioral repertoire in his or her natural context (see e.g., Mehl et al., 2001). Compared with elicited behavior, spontaneous behavior has the advantage of occurring in a natural context and natural situation, so it is not influenced by extraneous variables such as a non-natural context or social desirability based on researchers' expectations. However, this area of study remains highly complex, particularly when it comes to obtaining quantitative indicators that make it possible to reconstruct the “who,” “what,” “how,” and “when” of events of interest and position these events in the individuals' ecological niche. The difficult task of obtaining quantitative indicators of spontaneous behavior in everyday contexts has been further complicated by the long-standing clash between the qualitative and quantitative paradigms in psychology. Mixed methods research (Johnson et al., 2007) has provided valuable resources for combining qualitative data derived from non-spontaneous behavior (e.g., questionnaire responses) and narrative data derived from natural settings (e.g., a life story). Nevertheless, the merging of qualitative and quantitative perspectives in cases where only spontaneous behavior is of interest has been little explored. In this article, we describe a mixed methods approach grounded in observational methodology (Anguera, 2003) that we believe fills this gap. The proposed approach combines the strengths and offsets the weaknesses of the qualitative and quantitative perspectives.

We present a methodological framework for studying everyday behavior using a rigorous scientific approach based on indirect observation that involves “liquefying” transcribed verbal material or texts from original settings. The process involves the quantification of qualitative data using techniques that are based on order or sequence of events rather than on traditional frequency measures. The approach proposed is perfectly compatible with any guiding theoretical framework whatsoever; this method is not linked to any concrete theoretical model, it offers numerous methodological opportunities, and it has the potential to lead to significant developments in the field of studying everyday behaviors. This approach differs from previous work in this area (Sandelowski, 2001; Sandelowski et al., 2009; Seltzer-Kelly et al., 2012; Bell et al., 2016) in that it analyzes the order and sequence of events. The parameters of frequency (which only indicates the number of occurrences), order (which also provides information about sequence), and duration (which, in addition to the aforementioned information, also indicates the time in conventional units) provide a higher degree of data consistency (Bakeman, 1978). The use of the order parameter, with the introduction of sequentiality, entails added value of extraordinary importance (Sackett, 1980, 1987; Bakeman and Gottman, 1987; Magnusson, 1996, 2000, 2005, 2016; Sánchez-Algarra and Anguera, 2013; Portell et al., 2015a).

The presented liquefying method enables the systematic analysis of minor details that arise in a multitude of situations involving text (e.g., conversations, speeches, diary, or blog entries) with a level of granularity (Schegloff, 2000) that enables these “natural texts” to be analyzed in combination with other contextual data. The approach is applicable to both conventional and new forms of communication (e.g., WhatsApp messages), regardless of format or source. The source may be verbal behavior (informal conversations, focus group discussions, etc.) or documentary material (diaries, narratives, etc.), including in some cases graphic material, such as photographs and drawings.

Most of the solutions proposed to date for transforming text into quantitative data are either qualitative (e.g., ethnographic methods) or quantitative. Our proposal, however, takes a mixed methods approach in which spontaneously generated qualitative material is transformed into quantifiable code matrices.

In this article, we discuss key aspects of our proposed system. We analyze the concepts and meaning of systematic observation and one of its two branches, indirect observation, alongside key concepts of mixed methods research. We also look at types of qualitative data used in indirect observation and describe a methodological framework for building *ad hoc* observation instruments, creating matrices of codes for the data collected, and analyzing data and checking their reliability. Finally, we present a protocol specifically designed for indirect observation with examples from each of the stages in the process.

## From systematic to indirect observation

Psychologists work in a wide range of fields and subfields that correspond to everyday life situations. To name just a few examples, they are involved in health education programs in nurseries and nursing homes, prosocial programs in primary schools, exercise programs for the elderly, social support programs in neighborhoods, or communities with families of multiple nationalities, AIDS prevention programs for adolescents, support programs for families with a history of child abuse or negligence or families of young car crash victims, relaxation programs for athletes, and social programs in prisons or juvenile correctional institutions. Systematic observation can make important contributions to the study of spontaneous behavior in a vast range of everyday contexts.

Observation is a useful method for collecting, processing, and analyzing information that cannot be studied in the artificial setting of a laboratory. It enables a largely unbiased analysis of everyday behaviors and interactions that occur naturally (Anguera, 2010). Although systematic observation dates back to the 1970s, it has taken on an identity of its own in the last two decades (Anguera, 1979, 2003; Anguera and Izquierdo, 2006; Sánchez-Algarra and Anguera, 2013). It offers both flexibility and rigor as it is built on sound scientific principles, and this combination makes it ideal for use in many fields (Portell et al., 2015b).

Systematic observation differs from ethnography in that its purpose is not to obtain a narrative account of subjective experiences in a process that requires the participation of the researcher or person being studied. Ethnographic studies require a qualitative approach, but unlike systematic observation, they do not require quantitative analysis and rigorous data quality control. Systematic observation, by contrast, is characterized by highly systematic data collection and analysis, stringent data quality controls, and the merging of qualitative and quantitative methods.

Systematic observation follows the four fundamental stages of scientific research: formulation of a research question, collection of data, analysis of data, and interpretation of results. The wealth of data collected in an observational study provides researchers with the opportunity to capture valuable chunks or snippets of everyday realities, without having to specifically ask for the information (there are no interviews, questionnaires, or psychological tests). In addition, it allows the researcher to study spontaneous behavior in a natural, uncontrolled environment.

Everyday activity in context is the cornerstone of observational studies. It is the source of a rich fabric of information that the psychologist/researcher needs to tap into in order to extract relevant information that is subsequently processed systematically to produce a set of “net” data that can be analyzed both qualitatively and quantitatively.

The study of everyday activity provides insights into the diverse behaviors and events that occur throughout a person's life. It provides thus a privileged vantage point from which to observe changes, but everyday life is a highly complex, dynamic process replete with information that is often not even known to exist (Anguera, 2001). Its study requires the examination of diverse phenomena at different levels of a pyramid-like structure. At the top of the pyramid, psychologists analyze how individuals go about their lives and gradually become familiar with what has shaped their life course. As they move down the pyramid, they discover everyday realities at different levels (family, career, social relationships, hobbies, etc.) and come to understand how these are influenced by interacting factors, such as health, satisfaction of needs, and conflicts.

According to Mucchielli (1974) observation equation, O = P+I+Pk−B, observation equals perception plus interpretation plus previous knowledge minus bias. Observation thus is not possible unless what is being observed is perceivable. Perceptibility is a key concept when it comes to differentiating between direct and indirect observation (Anguera, 1979, 2003). In indirect observation, it is always incomplete, and Mucchielli's equation is only partially fulfilled.

In direct observation, perceptibility is considered to be complete when what is being observed (whether *in situ* or through video or audio recordings) can be captured by visual or auditory senses. In anthropology, for example, the subfield concerned with the study of visual representations is known as *visual* rather than *observational* anthropology. Modern-day technology permits maximum levels of precision in visual and auditory perception (Escalera et al., 2009; Bautista et al., 2015) and minimizes the need for interpretation.

Although everyday contexts can take countless shapes and forms, the levels of response (or criteria or dimensions) that can be directly observed are similar. Facial expressions, for example, can be analyzed by software such as Face Reader, which can distinguish between facial and emotional mimicry. Gestures, in turn, which also have an important role in human communication (Holle et al., 2012; Mashal et al., 2012), even in children (Lederer and Battaglia, 2015) can be effectively analyzed using programs such as NEUROGES+ELAN (Lausberg and Sloetjes, 2009, 2016). Finally, vocal behavior (Russ et al., 2008) can be analyzed using sound analysis software. Non-verbal manifestations, or “expressiveness,” are interesting external indicators of a person's emotional state (Rodriguez et al., 2014), although adequate quality control is needed to reduce bias.

While aspects of human communication such as facial expressions, gestures, posture, and voice tone are fully perceivable through visual or auditory channels, they are frequently accompanied by verbal behavior, which has very different characteristics in terms of perceptibility. Indirect observation is an appropriate method for studying both verbal behavior and textual material, whether in the form of transcripts or original material produced by the participants in a study.

Verbal behavior transmits messages and both these and the channels through which they are transmitted can take many shapes and forms. Messages are analyzed differently depending on whether they are spoken or written. Written forms of expression (e.g., self-reports, diaries, biographies) are largely considered to be narratives. Narrative studies have been used in qualitative methodology for many years and have both strengths and shortcomings. One of their main strengths is their adaptability to very different situations and contexts. Narrative studies provide insights into a person's true nature and help to understand their experiences and needs (Riva et al., 2006). They have been used, for example, in a wide range of settings, such as secondary schools (García-Fariña, 2015; García-Fariña et al., 2016), high schools (Tronchoni et al., 2018), family gatherings (Gimeno et al., 2006), support groups for patients (Roustan et al., 2013), therapeutic interaction (Blanchet et al., 2005), and group therapy for adolescents (Arias-Pujol and Anguera, 2017). One of their shortcomings is that perceptibility is limited by the documentary nature of the texts, and it is not uncommon for different researchers to draw different conclusions from the same text.

Human communication does not simply refer to the transmission of information. It involves numerous aspects that vary according to content, the people transmitting or receiving the message, their relationship (hierarchy, previous interactions, etc.), the flow of data or metadata, and the interpretative context. In addition, changing lifestyle habits and new technologies have led to new forms of human communication (Bavelas and Chovil, 2000), such as WhatsApp messages and blog posts, extending the traditional dichotomy between verbal and non-verbal behavior established by the classical sociologist Weick (1968, 1985). In a recent study, for example, Radzikowski et al. (2016) analyzed Twitter messages in a quantitative study on the rubella vaccine, and as stated by Hardley (2014, p. 34), “Over many decades, surveillance methods (often termed “indicator based” methods) have been developed and refined to provide disciplined, standardized approaches to acquiring and recording important information. More recently, ubiquitous and unstandardized data collected from the Internet have been used to gain insight into emerging disease events.”

Indirect observation can be considered a valid scientific method (Webb et al., 1966; Anguera, 1991, 2017, in press; Behar, 1993; Morales-Ortiz, 1999; Morales-Sánchez et al., 2014). It uses similar techniques to systematic observation, and as a procedure, it is structurally identical, although there are important differences dictated by the nature of the source data (verbal behavior and text).

Indirect observation involves the analysis of textual material generated either indirectly from transcriptions of audio recordings of verbal behavior in natural settings (e.g., conversation, group discussions) or directly from narratives (e.g., letters of complaint, tweets, forum posts). The addition of seemingly unobtrusive objects can also provide important insights into daily routines. All these materials constitute an extremely rich source of information for studying everyday life, and they are continuously growing with the burgeoning of new technologies for data recording, dissemination, and storage (Morales-Ortiz, 1999; Morales-Sánchez et al., 2014).

Narratives are an excellent vehicle for studying everyday life through indirect observation, and one option for studying them is to apply a procedure for systematizing and structuring the information through *quantitization*. This approach makes it possible to integrate qualitative and quantitative elements.

The data used in indirect observation invariably start out as qualitative and the source material varies according to the level of participation of the person being observed and the nature of the source (textual or non-textual).

Common sources of material used in indirect observation studies include:

- Recordings of verbal behavior as it occurs (normally in mp3 files). There may be single or multiple dialogues and it is essential to clearly distinguish between the different “voices” recorded.
- Transcripts of audio recordings of verbal behavior in a natural setting (Krueger and Casey, 2009). These may involve an individual (speaking, for example, in person or on the telephone), or a group (dyad, triad, focus group, etc.), in which each person can be clearly identified.
- Written texts produced by the participants in a research study. These include texts produced by the participants or those close to them (e.g., letters of complaint, letters to a newspaper, tweets, ads, messages on a mural, instant text messages). A variety of communication channels are possible (e.g., paper, e-mail, WhatsApp).
- Texts transmitted through the Internet, such as e-mails (Björk et al., 2014) and forum posts (Vaimberg, 2010). These constitute an extremely rich source of information and are particularly relevant to psychological interventions.
- Everyday objects related to the research question(s). While objects may appear to have a secondary role in communication, they can provide relevant insights into everyday life as they evoke or facilitate the expression of emotions through micro-valences (Lebrecht et al., 2012). Examples are graphs, paintings, models, and clay figures. Technological advances have also opened up new opportunities in this area in recent years.
- Graphic material, particularly photographs. These can constitute an extremely rich source of information (Zaros, 2016). A single photograph captures a moment, something static, but a gallery of photographs separated in time can capture the dynamics of an episode or successive episodes in the life of a person, or even a group or institution. This material can be primary (the only source available) or secondary (complementing other sources).
- Unobtrusive objects, also referred to as aggregates (Webb et al., 1966). These may simply be anecdotal, but in some cases they can reveal the existence of certain behaviors, but only after a process of inference involving variable risk. Examples are fingerprints and objects such as cigarette butts or a napkin with notes or drawings left behind in a café.

The above sources of information give rise to a varied set of data that provides empirical evidence and can position specific events and everyday behaviors along a continuum of time. Finally, the information available becomes progressively richer as one gains access to several sources of documentary material.

As mentioned, the material used to collect data in indirect observation is only partly perceivable (Anguera, 1991) and any conclusions made need to be inferred by a researcher drawing from a theoretical framework or taking a position. This is the main challenge in indirect observation. In the system we propose, rigorous application of a carefully designed observation instrument by duly trained observers offers the necessary guarantees of data reliability. Although direct and indirect observation may vary in terms of source material, level of interpretation, and level of participation, the two methods share a scientific procedure that when properly applied can provide quantitative indicators of the processes underlying everyday behavior.

## The challenges of mixed methods research

Mixed methods research has been increasingly embraced by the scientific community over the past 15 years (Creswell et al., 2003; Johnson et al., 2007; Tashakkori and Teddlie, 2010; Onwuegbuzie and Hitchcock, 2015). The mixed methods approach involves the collection, analysis, and interpretation of qualitative and quantitative data for the same purpose and within the framework of the same study; some authors have even raised the approach to the rank of paradigm. Molina-Azorín and Cameron (2015) acknowledge that mixed methods research is not easy to conduct and requires considerable time and resources. Nonetheless, it is a movement that is gradually gaining supporters. As stated by Leech and Onwuegbuzie (2009) and Onwuegbuzie (2003), mixed methods research lies on a continuum between single-method and fully mixed studies, although the scientific community has yet to agree on which position it holds along this continuum. That said, it is generally agreed that the position will depend on the research objective and the nature of the data, analyses, and level of inference.

Overall, mixed research is largely understood as “a synthesis that includes ideas from qualitative and quantitative research” (Johnson et al., 2007, p. 113). However, this is a very broad framework in which many gaps need to be filled. In the case of indirect observation, the methodological approach must be extremely rigorous as we are dealing with situations in which substantive areas merge with the multiple realities of everyday life.

The exponential growth of mixed methods research in recent decades has generated certain inconsistencies in terms of terminology and definitions. We therefore believe that it is first necessary to clarify the meaning of method/methodology and to discuss the multiple meanings attached to the term “mixed method” before we present our methodological framework for indirect observation.

Greene (2006, p. 93) proposed a broad description of the term “methodology,” understood as an inquiry logic that admits different forms of data collection (questionnaires, interviews, observational datasets, etc.), methods of research (experimental, ethnographic, etc.), and related philosophical issues (ontology, epistemology, axiology, etc.). Greene also refers to specific guidelines for practice, which distinguish between methods that obviously vary in terms of design, sampling, data gathering, analysis, etc. We consider that systematic observation fits with Greene's definition of methodology (Anguera, 2003), although we have not always used the term. We also agree with the following statement by Johnson et al. (2007, p. 118): “It is important to keep in one's mind, however, that the word *methods* should be viewed broadly.” Accordingly, in the approach we describe in this article, we also consider indirect observation to be a method in the broad sense of the word.

Johnson et al. (2007, p. 123) defined mixed methods research as “the type of research in which a researcher or team of researchers combines elements of qualitative and quantitative research approaches (e.g., use of qualitative and quantitative viewpoints, data collection, analysis, inference techniques) for the broad purposes of breadth and depth of understanding and corroboration” (Johnson et al., 2007, p. 123). They formulated this definition after asking 19 renowned researchers in the field (Pat Bazeley, Valerie Caracelli, Huey Chen, John Creswell, Steve Currall, Marvin Formosa, Jennifer Greene, Al Hunter, Burke Johnsson and Anthony Onwuegbuzie, Udo Kelle, Donna Mertens, Steven Miller, Janice Morse, Isadore Newman, Michael Q. Patton, Hallie Preskill, Margarete Sandelowski, Lyn Shulha, Abbas Tashakkori, and Charles Teddlie) to send in their definition of the term “mixed methods” by e-mail.

We fully agree with the definition proposed by Johnson et al. (2007) and it provided us with the necessary elements to draw up our methodological framework for indirect observation. The success of any mixed methods approach depends on the adequate mixing or integration of qualitative and quantitative elements. Numerous authors have analyzed the term “mixing” in an attempt to provide guidance on the processes required to achieve a seamless result (Bazeley, 2009; O'Cathain et al., 2010; Fetters and Freshwater, 2015). Qualitative and quantitative data can be mixed in three different ways, aptly summed up by Creswell and Plano Clark (2007, p. 7): “There are three ways in which mixing occurs: merging or converging the two datasets by actually bringing them together, connecting the two datasets by having one build on the other, or embedding one data set within the other so that one type of data provides a supportive role for the other data set.” For our proposal, we chose the second form: connecting two databases by having one build on the other. According to Sandelowski et al. (2009), this connection can be achieved through transformation, i.e., by quantitizing qualitative data or qualitizing quantitative data. In our indirect observation framework, we transform non-systematic qualitative data into a format suitable for quantitative analysis.

Mixed methods research is marked by a persistent scientific gap that requires powerful solutions rooted in two key challenges in the field of indirect observation. These two challenges, discussed in this article, are (a) how to rigorously transform qualitative textual material derived largely from everyday human communication into matrices of codes, and (b) how to subsequently analyze these codes using quantitative methods suited to the categorical nature of the data in order to uncover the underlying structure. The proposed transformation system breaks away from the classical theoretical framework of mixed methods, which simply involves integrating qualitative and quantitative elements. The key difference is that it contemplates systematic observation, and hence indirect observation, to be a mixed method in itself (Anguera and Hernández-Mendo, 2016; Anguera et al., 2017a).

Integration of qualitative and quantitative elements is the key to any mixed methods approach (Creswell and Plano Clark, 2007; Bazeley, 2009; O'Cathain et al., 2010; Maxwell et al., 2015). Our approach adds another element: the liquefaction of verbal behavior and texts. This process consists of schematically transforming “solid” textual material into “liquid” matrices of codes apt for quantitative analysis (Anguera et al., 2017b; Anguera, in press). The quantitative processing of originally qualitative data with the aim of detecting hidden behavioral patterns or underlying structures, for example, adds an element of robustness to the integration of qualitative and quantitative data, particularly in the case of everyday life events and behaviors.

Talkativeness and text, for example, can now be analyzed within the framework of mixed methods research using frequency counts (Poitras et al., 2015) thanks to the development of reliable—and extremely useful—measures of verbal productivity and the multiple opportunities offered by modern-day technology (Bazeley, 2003, 2006, 2009). Frequency counts, however, are weak and insufficient measures. Considering that “methodological plenitude” (Love, 2006, p. 455) is not always attainable in applied research, the mixed method framework offers new and interesting possibilities for indirect observation.

The combined use of qualitative and quantitative approaches has been tried and tested in multiple studies and has also been analyzed in several systematic reviews (Elvish et al., 2013). In the following sections, we show that it is necessary to start with qualitative inputs and to then quantify these in a process that ensures reliability throughout the various stages.

## Qualitative datasets in indirect observation

The empirical process in indirect observation starts with the collection of qualitative data. While the characteristics and standards that guarantee quality are perfectly outlined in the literature on quantitative methodology, the same cannot be said of qualitative methodology. Qualitative methodology offers enormous flexibility, but interpretations on content and form vary and are not free of controversy. Content provides personal and interpersonal information, which stems from experiences that are temporally unstable and highly influenced by the context and versatility of the moment. As for form, the tools used to support indirect observation (narratives, biographies, self-reports, life stories, in-depth interviews, etc.) cause doubt and distrust in many researchers, who, in the absence of standardized tools, question their stability and consistency.

Much has been written about the forms used to structure narratives (e.g., Hurwitz et al., 2004; De Fina and Georgakopoulo, 2015; Riessman, 2015), and qualitative data can be gathered using many tools, including interviews (e.g., Riera et al., 2015), biographies (e.g., Lindqvist et al., 2014), children's vignettes (e.g., Jackson et al., 2015), focus group vignettes (e.g., Brondani et al., 2008), telephone interviews (e.g., Björk et al., 2014), self-reports (e.g., Coutinho et al., 2014), focus group recordings (e.g., McLean et al., 2011), and participant observation (e.g., Caddick et al., 2015). In our case we are specifically interested in qualitative datasets within the framework of indirect observation. Although systematic observation dates back to the 1970s, it has taken on an identity of its own in the last two decades (Anguera, 2003; Anguera and Izquierdo, 2006; Sánchez-Algarra and Anguera, 2013; Anguera et al., 2017a). Indirect observation shares many of the characteristics previously described for systematic observation, namely, highly systematic data collection and analysis, strict data quality controls, and an approach that requires the merging of qualitative and quantitative techniques.

## A methodological framework for liquefying text

In these next sections, we are going to describe, and illustrate with examples, the stages and sub-stages involved in an indirect observation study. We will focus largely on the extraction and transformation of information from textual material produced using conventional or newer channels of communication in a variety of formats (handwritten letters, reports, transcriptions of group meetings, and interviews, etc.), irrespective of origin (e.g., informal conversations or focus group discussions or documentary material).

Extracting information on human behavior from text and transforming it into suitably systematized and organized categorical data, without loss of key information, is a major challenge in the Behavioral Sciences. In addition, the process must offer sufficient scientific and ethical guarantees and produce results in a format that can be rigorously processed using any of a range of quantitative techniques available for analyzing categorical data.

Our text-liquefying process consists of six stages: (1) specification of study dimensions, (2) establishment of segmentation criteria to divide the text into meaningful units, (3) building of a purpose-designed observation instrument, (4) coding of information, (5) data quality control, and (6) quantitative analysis of data. **Table 9** presents detailed steps and guidelines for the “liquefication” of indirect observations. Each of the steps will be explicated within the following sections.

### Specification of study dimensions

In systematic observation, and by extension, indirect observation, the term “dimension,” also known as level of response (Weick, 1968) or criterion, refers to a distinguishable facet related to the research objective. Dimensions are generally derived from a theoretical framework (e.g., the seminal work of (Weick, 1985) in the field of social interaction), but they can also be created *ex novo* based on experience or expertise. In the latter case, they must always be justified.

Studies can be one-dimensional or multidimensional. It is not uncommon for researchers to start off with a single dimension and then gradually add others as they delve deeper into the theoretical framework. Below are examples of dimensions and theoretical frameworks used in three indirect observation studies. In the first case, a study of disruptive behavior and communication difficulties in adolescents participating in group communication therapy, Arias-Pujol and Anguera (2004) proposed the dimensions verbal and non-verbal behavior, derived from the corresponding interpersonal theoretical framework (Danzinger, 1982; Gale, 1991; Poyatos, 1993). In the second case, Vaimberg (2010), on studying a psychotherapy group in which participants were able to write what they wanted on an online forum at any time over 3 years, chose the following dimensions: in-person, otherness, emotionality, thoughtfulness, positivity, and realism. The theoretical framework was built from work by various authors (e.g., Winniccott, 1979; Bion, 1985; McDougall, 1991; Lévy, 1995). In the third case, which was a recent study of teacher-led discourse in physical education built on the theoretical framework of the Teaching Games for Understanding model (originally proposed by Bunker and Thorpe, 1982) and work on discourse strategies by Coll and Onrubia (2001), García-Fariña et al. (2016) proposed nine dimensions: exploration and activation of previous knowledge, attribution of positive meaning by students, progressive establishment of increasingly expert and complex representations of subject matter, interactivity segment, message structure, extralinguistic resources, task type, destination of message, and location of session.

### Specification of segmentation criteria to create textual units

The second step toward liquefying a text is to define the segmentation criteria to divide the text into meaningful units. This process is known as “unitizing.” Although initially proposed by Dickman (1963) and Birdwhistell (1970), Krippendorff (2013, p. 84) defined unitizing as “the systematic distinctions with a continuum of otherwise undifferentiated text—documents, images, voices, websites, and other observables—that are of interest to an analysis, omitting irrelevant matter but keeping together what cannot be divided without loss of meaning.” This definition suggests that it would be logical to first segment the text into primary criteria within the main study dimension and then establish secondary criteria for the other dimensions (e.g., voices, gestural behavior, etc.).

Krippendorff (2013) suggested segmenting text using orthographic, syntactic, contextual, and inter-speaker criteria. In this last case, each intervention by an individual is considered a unit. This is a very useful approach for analyzing interactions between various people. We propose using the inter-speaker criterion as the primary criterion and subsequently establishing secondary criteria (subunits) for verbal or written interventions containing various syntactic elements (phrases).

In cases with several dimensions, such as verbal behavior accompanied by gestures, postures, or exchange of looks, verbal behavior, as the most perceivable behavior, could be established as the primary criterion. The other behaviors could then be segmented into subunits as appropriate. In very specialized cases, however, we consider that the above level of segmentation is insufficient. The initial segmentation stage is crucial as the categories that will be created in the next stage will directly determine the content of the dataset for analysis. Where possible, test runs or pilot studies should be performed first. Table 1 shows how a conversation between two anonymous speakers is segmented into units.

**Table 1:** Vignette showing the segmentation of a text (transcribed from a conversation) into units.

S1. The truth is that I sometimes doubt whether I like basketball that much [U1], even though I have already devoted 15 years of my life to the game [U2].
S2. But you started as a young boy [U3], when you were given the possibility of playing as a junior at school [U4].
S1. That moment was very important for me [U5], as I got carried away with the enthusiasm [U6] and I couldn't go for a day without playing [U7]. Then, when I finished secondary school, I got the opportunity to join the club where I am now [U8] and to dedicate myself in body and soul to basketball [U9].
S2. Did you think back then about what this decision would entail? [U10].
S1. I couldn't tell you exactly…[U11] I think I was somewhat confused [U12], as on the one hand I wanted to study industrial engineering, probably influenced by my father and my uncle [U13], but on the other, the fact that I was valued, without being particularly tall [11], was a golden dream [U15]. I think that I was living between real life and the dream…[U16] And I accepted straight away [U17], although after talking it through with my parents, uncle and brother [U18]. They gave me some opinions and advice [U19], but left the final decision up to me [U20].

### Building an indirect observation instrument

Indirect observation studies, like systematic observation studies (Anguera, 2003; Anguera and Izquierdo, 2006; Sánchez-Algarra and Anguera, 2013; Portell et al., 2015a) require a purpose-built observation instrument to systematically code the information that will form the subsequent datasets.

Observation instruments can be built using category systems, a field format system, a combination of these systems, or rating scales (Anguera et al., 2007). One-dimensional studies use category systems and rating scales, while multidimensional studies use field formats or field formats combined with category systems. To build a category system, there must be a theoretical framework, and to build a rating scale, it must be possible to grade the corresponding dimensions ordinally. In addition, the category system must fulfill the requirements of exhaustivity and mutual exclusion, and each category must be accurately defined.

The field format is built by creating a catalog of mutually exclusive behaviors for each dimension. As it is not exhaustive, the catalog is left open and is therefore considered to be in a permanent state of construction. While not required, a theoretical framework is recommendable for field format systems.

Observation instruments combining a field format system with category systems are becoming increasingly common. This combination is possible when some or all of the dimensions in the field format have a theoretical framework and the object of research is atemporal (i.e., it is not a process).

To simplify matters, it is highly recommendable to code both categories and dimensions using letters, numbers, or symbols. If A, B, C, and D are categories in a category system, i.e., fulfilling the requirements of exhaustivity and mutual exclusion (e.g., A = XX, B = XX, C = XX, and D = XX), then the notation would be CS (category system) = {A B C D}. If A, B, C, and D are behaviors in an open catalog, i.e., they are mutually exclusive but not exhaustive (e.g., A = XX, B = XX, C = XX, and D = XX), the notation would be Catalogue = A B C D…

### Guidelines for coding information

Observational datasets created from narratives (Crawford, 1992; Gabriel, 2004; Tuttas, 2015) have wide applications in many everyday life situations. However, before qualitative inputs from human communication can be transformed into quantitative data, it is first necessary to decide how to organize the heterogeneous information available. This process can be extremely complex as it is necessary to bring together data from very different sources, and very possibly, different points in time (Duran et al., 2007). The first step is to correctly record and code the data, and this is where the *ad hoc* observation instrument becomes invaluable. As started by Bradley et al. (2007, p. 1,761), “coding provides the analyst with a formal system to organize the data, uncovering and documenting additional links within and between concepts and experiences described in the data.”

If the sources have been carefully selected, they will all contribute to creating a stockpile of information on the behaviors or actions of all those involved in the communication process being analyzed (e.g., therapists, participants, supervisors…).

The system for processing narratives or bodies of texts is quite similar to that used in discourse analysis (Calsamiglia and Tusón, 1999), although the information retrieved is richer and more diverse. Once the necessary quality controls are in place, the information can be managed and processed systematically within an empirical research setting that ensures replicability. Examples of texts used for this purpose are interviews, speeches, and conversations (Sidnell and Stivers, 2013). These may be a specific audience, a single speaker or several (with turn-taking), words in isolation, or, when direct and indirect observation are combined, words accompanied by tone/pitch, gestures, facial expressions, posture, objects, etc (Fischer et al., 2012).

Once the study dimensions have been selected (section Specification of Study Dimensions) and the text has been segmented into units (section Specification of Segmentation Criteria to Create Textual Units) and the behaviors coded using the *ad hoc* observation instrument (section Building an Indirect Observation Instrument), the data can be transformed into a series of complete or incomplete code matrices containing purely qualitative information (Anguera, 2017, in press; Anguera et al., 2017b). This transformation is achieved by organizing the dimensions into columns and adding the behavioral units to the corresponding rows, achieving thus a “liquid” text, ready for quantitative analysis (Table 2 contains an example).

**Table 2:** Tabular structure for creating a code matrix.

		**Dimensions (i)**			
		**D1 [Verbal behavior]**	**D2 [Vocal behavior]**	**…**	**Di**
**Textual units (n)**	**U1 [Initial greeting]**	Code … [Informal expressions]	Code … [Raised voice with laughter]		Code …
	**U2 [Activity to carry out]**	Code … [We have to solve the math problems]	Code … [Normal voice]		
	**…**				
	**Un**	Code …	Code …	…	Code …

*Each row of the matrix contains a series of boxes that are completed with codes corresponding to each textual unit (fragments of text from indirect observation). The columns, in turn, contain the different dimensions, or criteria, of the observation instrument. The codes come from the ad hoc observation instrument and may correspond to behaviors from a field format catalog or to categories in an observation instrument based on category systems only or on category systems combined with a field format. By way of illustration, we have added in brackets the first two dimensions (verbal behavior and vocal behavior), a simulation of the first units and an indication of the behaviors produced (which will be coded)*.

Table 3a shows a hypothetical example of data extracted from a text in a one-dimensional study using an observation instrument with category systems, using a simulated example of the diary of a patient with endogenous depression. Table 3b, in turn, shows the results for a combined field format-category system instrument from a multidimensional study, using a simulated example of an oral mediation situation involving a conflict between the parties A and B, with the assistance of the mediator C. These matrices of codes (Table 3a is atypical as it has just one column due to the single dimension analyzed) show how the qualitative data have been structured.

**Table 3:** **(a,b)** Hypothetical examples of a code matrix derived from a text.

**a**	**b**
Diary of a patient diagnosed with endogenous depression: SC = {A B C D} A: Expressions of sorrow or sadness B: Expressions of self-perceived improvement C: Expressions of self-perceived worsening (situation of hopelessness) D: Expressions of joy at having overcome the problem [This is an exhaustive and mutually exclusive system of categories, constructed from a theoretical framework (Altimir et al., 2010; Dagnino et al., 2012; Krause et al., 2016)] **Table:** **Units** **Categ**. U1 A U2 B U3 D U4 A U5 C U6 A U7 B U8 D U9 A U10 D U11 A U12 B U13 C U14 A U15 B U16 D U17 A U18 B U19 D U20 C	Oral mediation situation involving a conflict between the parties A and B, with the assistance of the mediator C: E = {E1 E2 E3 E4 E5} F = F1 F2 F3 F4 F5 F6 F7 F8 … G = {G1 G2 G3} H = H1 H2 H3 H4 … Dimension E: Verbal behavior E1: Facilitating elements (greeting, courtesy routines, etc.) E2: Focused on the crux of the issue E3: Related to secondary aspects E4: Neutral sentences not related to the conflict E5: Conflictive elements (insults, mockery, etc.) [This is an exhaustive and mutually exclusive system of categories] Dimension F: Vocal conduct F1: Shouting F2: Speaking in an annoyed tone F3: Speaking loudly F4: Speaking while crying F5: Speaking normally F6: Speaking softly F7: Whispering F8: Silence [This is a catalog of behaviors; as such, it is an open list and additional codes can be added] Dimension G: Interacting parties G1: Party A G2: Party B G3: Mediator [This is an exhaustive and mutually exclusive system of categories] Dimension H: Expression of displeasure/disagreement H1: Shaking head to indicate “no” H2: H1 plus hands clasped H3: H2 plus bulging eyes H4: H3 plus clenched jaw [This is a catalog of behaviors; as such, it is an open list and additional codes can be added] **Table:** **Units** **Dim. E** **Dim. F** **Dim. G** **Dim. H** U1 E1 G1 H1 U2 E2 F5 G2 H3 U3 E2 F5 G2 H2 U4 E3 F5 G1 H2 U5 E3 F1 G2 U6 E3 F1 G2 U7 E5 F1 G3 U8 E5 F1 G1 U9 F5 G1 U10 F5 G1 H1 U11 E1 F5 G2 H2 U12 E1 G1 U13 E1 F4 G3 H1 U14 E2 F2 G3 U15 E2 F2 G3 H1

*The columns correspond to the dimensions and the rows to the units into which they were segmented. Codes on the same row reflect concurrent behaviors. The codes are defined in the ad hoc instrument designed for the study*.

Additional sources of information, such as drawings, sounds, or photographs can be incorporated simply by adding new dimensions. Although this is still a relatively new concept, it is perfectly feasible with today's advanced coding systems (Saldaña, 2013) and technological possibilities (e.g., Bazeley, 2003, 2006, 2009; Crutcher, 2003, 2007; Holtgraves and Han, 2007; Romero et al., 2007; Dam and Kaufmann, 2008; Taylor et al., 2015). In the ATLAS.ti (v.7) qualitative data analysis program, for example, the text coding feature can be used to supplement the information entered with an object or an audio or video recording.

Researchers now have access to a multitude of software programs that facilitate their work. For those working with indirect observation, the CAQDAS platform (AQUAD6, ATLAS.ti, MAXqda2, NUDIST, NVivo, etc.) offers numerous programs for segmenting and coding text, and there are also open-access programs, such as T-LAB (http://tlab.it/en/presentation.php), IRAMUTEQ (www.iramuteq.org), and those created by the Italian group GIAT (www.giat.org). Numerous considerations are necessary when extracting information from text using content analysis techniques. Content analysis programs have traditionally favored the processing of large, mostly qualitative, bodies of texts, graphs, and audio and video material. The analysis uncovers relational structures (families, networks, etc.) that are relatively stable, or at least appear to be, and are always determined by the choices of the researcher. Nowadays, however, powerful software programs can analyze multiple sources of information to produce code matrices (Vaimberg, 2010) that are of enormous value for analyzing human communication in many fields.

Two programs can be used for both direct and indirect observation. These are HOISAN (Hernández-Mendo et al., 2012) (http://www.menpas.com), which is open-access and is available in several languages (English, Spanish, Portuguese, French) that can be selected from the tab *Archivos (Files)*, and TRANSANA (http://www.transana.com).

## Quantitative processing of code matrices

### Rigorous data quality control

The issue of data quality in indirect observation has been widely debated in the literature, with a particular focus on reliability and validity, and concerns have led many psychologists and researchers working in this area to modify their approaches. Both intraobserver and interobserver agreement are important measures of reliability, but they are not the only ones. While reliability is necessary, it alone does not guarantee the validity of a dataset (Krippendorff, 2013).

Krippendorff (2013) was the first author to insist on rigorous data quality control as a requirement for the quantification of data resulting from indirect observation. Thanks to his contributions in this area, there are now methodological tools in place to demonstrate the quality of such data. The two main quantitative measures for testing the reliability of data from direct observation (behaviors) and indirect observation (texts) are (a) coefficients of agreement between two observers who separately code behaviors using the same dataset and observation instrument and (b) coefficients of agreement based on correlation. Numerous coefficients exist for quantitatively verifying the quality of data in a wide range of situations. One widely used measure in indirect observation is Krippendorff's canonical agreement coefficient, which is an adaptation of Cohen's kappa coefficient for analyzing three or more datasets. It can be calculated in HOISAN. Another option for use in situations with different sources of variation is generalizability theory (Blanco-Villaseñor, 2001; Escolano-Pérez et al., 2017).

A more qualitative method, the consensus agreement method (Anguera, 1990), is gaining increasing recognition in indirect observation and other studies. In this method, at least three observers work together to discuss and agree on the most suitable code for each unit from the observation unit. This method has obvious advantages, as it produces a single dataset and frequently results in a better observation instrument thanks to the detection of possible gaps and shortcomings. While it offers significant guarantees of quality, however, it also carries risks. An observer may defer to the decisions of a more senior or “expert” colleague, for example, and the need to agree can also give rise to frictions or conflicts. The results of the consensus agreement method can be complemented by quantitative measures of agreement (Arana et al., 2016).

There has been much debate in the field of psychology about the extent to which adherence to a particular theoretical framework may influence agreement between observers. To overcome this potential problem, Pope et al. (2000) proposed using observers from different backgrounds to analyze the data. Such an approach, however, would require even more rigorous quality control measures given the greater difficulty of reaching agreement.

Table 4 shows the canonical agreement coefficient calculated in HOISAN for the data in Table 3b, combined with two other sets of data recorded for the same section of text by the same observer and with the same instrument, but at different moments.

**Table 4:** Example of datasets used to calculate intraobserver canonical agreement.

**Occasion 1**	**Occasion 2**	**Occasion 3**	**Canonical agreement**
E1 G1 H3	E1 G1 H3	E1 G1 H3	0.84
E2 F5 G2 H3	E2 F5 G2 H3	E2 F5 G2 H3	Result: Satisfactory agreement
E2 F5 G2 H2	E2 F5 G2 H2	E3 F5 G2 H2	
E3 F5 G1 H2	E3 F5 G1 H2	E3 F5 G1 H2	
E3 F1 G2 H4	E3 G2 H4	E3 F1 G2 H4	
E3 F1 G2	E3 F1 G2	E3 F1	
E5 F1 G3	E5 F1 G3	E5 F1 G3	
E5 F1 G1	E1 F1 G1	E1 F1 G1	
F5 G1	F5 G1	F5 G1	
F5 G1 H1	F5 G1 H1	F5 G1 H1	
E1 F5 G2 H1	E1 F5 G2 H1	E1 F5 G2 H1	
E1 G1	E1 G1	E1 G1	

*In such cases, the same verbal behavior or textual material must be coded by the same observer, using the same indirect observation instrument, on three separate occasions, separated by at least a week. The data in the first column are from Table 3b*.

### Quantitative processing of code matrices

Once the text has been liquefied and the necessary data controls performed, the researcher now has access to a series of code matrices perfectly suited for analysis using different techniques.

The novel nature of our proposal is that we do not study frequency counts, which, despite their serious limitations, were the only measure of quantification used in observation studies for decades.

Over the last 15 years, our group has prioritized three analytical techniques that are particularly well-suited to processing qualitative data in both systematic observation (Blanco-Villaseñor et al., 2003) and indirect observation studies. These are lag sequential analysis, polar coordinate analysis, and T-pattern detection. All three techniques are based on statistical calculations and therefore provide the necessary guarantees of replicability and robustness.

#### Lag sequential analysis

Lag sequential analysis, which works with code matrices (see example in Table 5), is used to detect behavioral patterns that show the structure of interactive episodes (Bakeman, 1978, 1991; Bakeman and Gottman, 1987; Bakeman and Quera, 1996, 2011). The analysis can be performed prospectively (looking forward in time from a given moment) or retrospectively (looking backwards) using positive or negative lag counts. A behavior, for example, with a lag count of +2 would correspond to a behavior that occurs 2 positions after the behavior(s) of interest, while one with a lag count of −2 would correspond to a behavior that occurs 2 positions before the behavior(s) of interest.

**Table 5:** **(a)** The first row shows the simple frequency counts for the data from Table 3a. The matrix below shows the transition frequencies for the given behavior A with the conditional behaviors shown at the head of each column. The different lags are shown by rows. **(b)** The first row shows the unconditional probabilities while the rows below show the conditional probabilities.

**a**						**b**			
**Lag**	**A**	**B**	**C**	**D**	**Total**	**A**	**B**	**C**	**D**
	7	5	3	5	20	0.35	0.25	0.15	0.25
1	0	5	1	1	7	0	**0.71**	0.14	0.14
2	2	0	1	4	7	0.28	0	0.14	**0.57**
3	4	2	1	0	7	**0.57**	**0.28**	0.14	0
4	0	2	2	2	6	0	**0.33**	**0.33**	**0.33**
5	4	0	0	1	5	**0.8**	0	0	0.2

*Bold values are significative (upper that respective unconditional probabilities)*.

The analysis can be applied to part of a session, to a complete session, to parts of different sessions (e.g., the first few minutes of a series of sessions), or to series of complete sessions. The technique thus offers enormous flexibility in terms of addressing different research questions. Two types of data can be used: data for which only the order of occurrence of concurrent behaviors has been recorded, using any of the free software programs available SDIS-GSEQ v. 4.1.2 (Bakeman and Quera, 2011), GSEQ5 (Bakeman and Quera, 2011), or HOISAN v. 1.6.3.3 (Hernández-Mendo et al., 2012), and data for which both order and duration have been recorded (SDIS-QSEQ and GSEQ5). Lag sequential analysis has been successfully applied in many indirect observation studies conducted over the past 25 years (e.g., Martínez del Pozo, 1993; Arias-Pujol and Anguera, 2004; Cuervo, 2014).

Using the data from Table 3a again, we illustrate how to manually calculate the results for the first, and simple, part of the lag sequential analysis process. The first step is to create tables for the matching frequencies and probabilities (Tables 5a,b) for category A (in our example, expressions of sorrow or sadness), which, according to the hypothesis applied, is the given behavior (the behavior of interest). In row 1, for example, A has a frequency count of 0 because this code does not occur again; B (expressions of self-perceived improvement) has a count of 5 because it occurs after A on five occasions (units 2, 7, 12, 15, and 18); C (expressions of self-perceived worsening) has a count of 1 because it only occurs after A on one occasion (unit 5), similarly to D (expressions of joy at having overcome the problem) (unit 10). In row 2, in turn, A has a count of 2 because it occurs on two occasions (units 6 and 11) in the second position after the given behaviors (units 4 and 9, respectively); B has a count of 0 because it does not occur in the second position after the given behavior; and C has a count of 1 because it occurs just once (unit 13) in the second position after the given behavior (unit 11), and so on.

The data are analyzed to search for behavioral patterns, with consideration of some or all of the other behaviors, known as target behaviors, to see if they form part of the pattern(s) detected.

**Figure 1:**
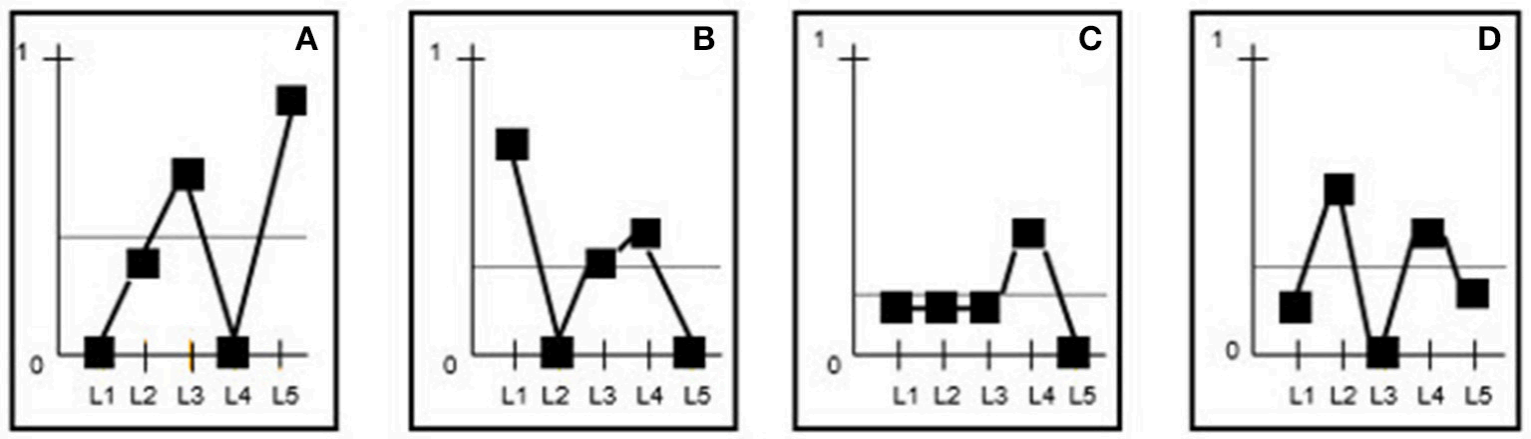
**(A–D)** The lags are shown on the X-axis and the probabilities on the Y-axis. Based on the results from Table 5b, the values corresponding to the unconditional probabilities (first row) are indicated by the horizontal line parallel to the X-axis (e.g., 0.35 for category A). Also shown are the values for each of the conditional probabilities for each category and lag. These values are linked by a (generally uneven) line for each category. The horizontal line parallel to the X-axis represents the upper limit for the effect of chance. Accordingly, any conditional probabilities in the subsequent lags that are higher than the unconditional probability for the corresponding category are significant and hence form part of the behavioral pattern.

The information for each of the categories is shown on a graph with the lags on the X-axis and the probability values (ranging between 0 and 1) on the Y-axis. Each of the four Figures 1A–D, shows the value of the unconditional probability (the line parallel to the Y-axis) and the points corresponding to the conditional probability of each lag.

**Figure 2:**
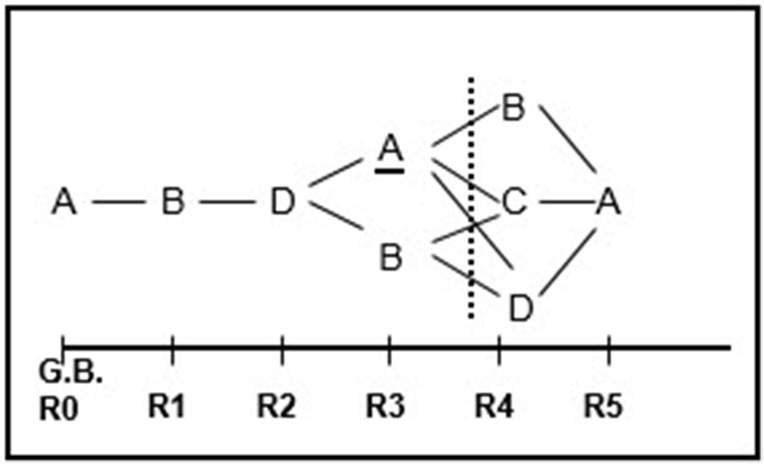
Behavioral pattern extracted after assigning significant conditional behaviors (behaviors with a conditional probability greater than the unconditional probability) to each lag. The behavior pattern extracted from the presented illustration exhibits a regularity consisting of expressions of sorrow or sadness being followed by expressions of self-perceived improvement and these expressions, in turn, being followed by joy at having overcome the problem. From there, the pattern bifurcates, leading either to the initial situation of sorrow and sadness or to expressions of self-perceived worsening.

Based on this simple visual output and considering all the statistically significant categories at each lag (i.e., the categories with a conditional probability value greater than that of the unconditional probability), we extracted the behavioral pattern shown in Figure 2. The strength of patterns is assessed using interpretative rules (Bakeman and Gottman, 1987). In the example provided, the first lag that is followed by another lag containing significant categories is considered to be the last lag (max lag) in the pattern (lag 3 in the example).

The robustness of the pattern must then be further strengthened by building a confidence interval around the conditional probabilities, for which only the upper limit is needed. This upper limit is used to determine whether a given category will form part of the pattern at the lag being analyzed, as the conditional probability obtained has to be higher than unconditional probability. The lower limit, by contrast, will always be lower than the unconditional probability and as such, will never be significant. Application of this confidence interval increases the requirements for statistical significance for the categories at each lag, resulting in a more robust corrected pattern.

The results obtained by applying the formula corresponding to the corrected expected or unconditional probability (shown in Table 6a) are presented in Table 6b, which is an extension of Table 5b.

**Table 6:** **(a)** Formula for calculating the corrected unconditional (expected) probability. **(b)** Table showing the probabilities from Table 5b with the addition of the corrected conditional probabilities in the second row (bold values).

**a**	**b**			
$\begin{array}{c} {\text{p}}_{\text{esp corr}}={\text{p}}_{\text{esp}}+{\text{Z}}_{\alpha }\sigma \\ \sigma =\sqrt{\frac{{\text{p}}_{\text{esp}}\left(1-{\text{p}}_{\text{esp}}\right)}{\text{N}}} \end{array}$	**A**	**B**	**C**	**D**
	0.35	0.25	0.15	0.25
	**0.56**	**0.43**	**0.31**	**0.43**
	0	**0.71**	0.14	0.14
	0.28	0	0.14	**0.57**
	**0.57**	**0.28**	0.14	0
	0	**0.33**	**0.33**	**0.33**
	**0.8**	0	0	0.2

*These correspond to the upper limit of the confidence interval built around the unconditional probability values, with p < 0.05*.

A second optimization step involving the calculation of adjusted residuals or hypergeometric *Z*-values (Allison and Liker, 1982) is also possible but cannot be done manually.

**Figure 3:**
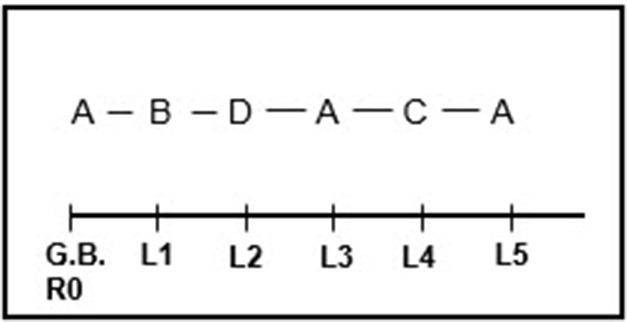
Optimized corrected behavioral pattern following construction of a confidence interval around the unconditional probabilities. The corrected pattern reveals the typical alternation seen in patients with endogenous depression.

**Figure 4:**
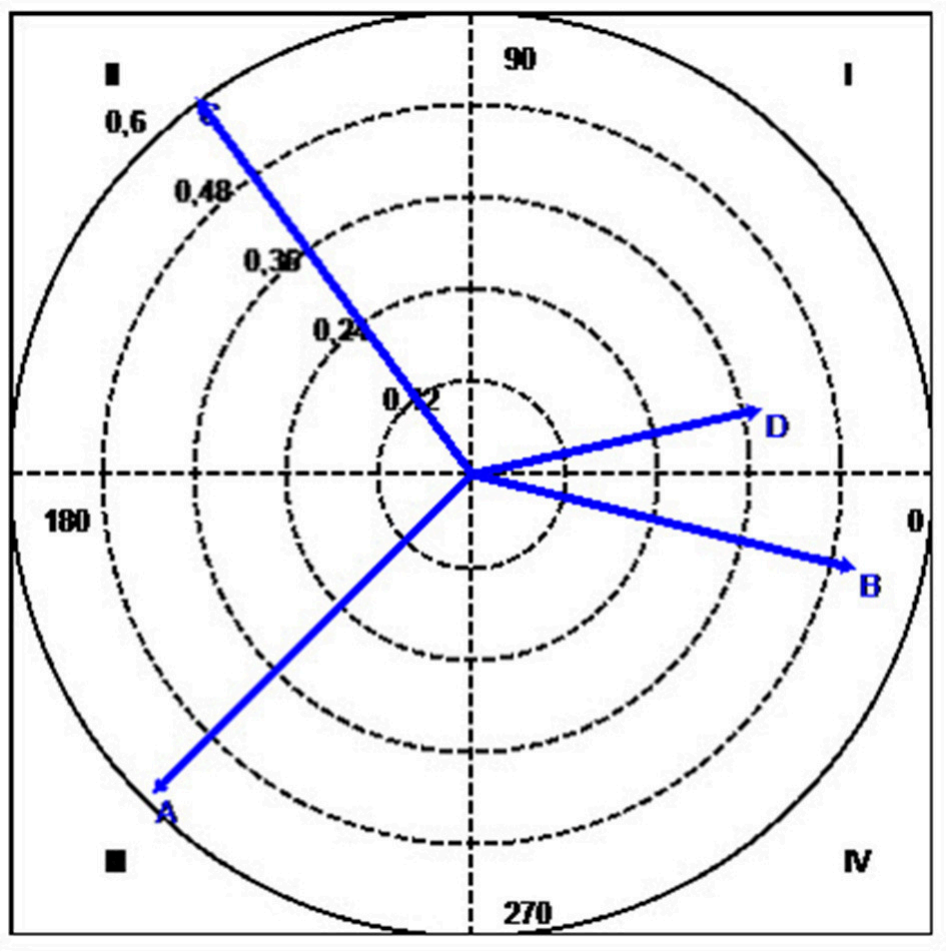
Polar coordinate map showing the vectors for the categories A (focal category), B, C, and D. As indicated in the legend of Table 8, A is the focal behavior and expressions of sorrow or sadness activate expressions of self-perceived improvement (Quadrant IV) and joy at having overcome the problem (Quadrant 1). The focal behavior is not self-generating (Quadrant III). Additionally, expressions of sorrow or sadness do not generate self-perceived worsening (Quadrant II), although self-perceived worsening does generate the focal behavior.

**Figure 4** shows the corrected behavioral pattern extracted from the data in Table 6b. As shown, it is different to the uncorrected pattern shown in Figure 3. Note that in both cases, A, the given behavior, is statistically associated with B at the first lag and D at the second lag.

Lag sequential analysis is the first of the three key techniques we use in our text-liquefying approach to indirect observation. It has been widely used in systematic observation studies from a range of areas published in journals listed in the Journal Citations Report (JCR) (e.g., Gimeno et al., 2006; Lapresa et al., 2013; Roustan et al., 2013).

#### Polar coordinate analysis

Polar coordinate analysis, which was proposed by Sackett (1980), combines adjusted residuals from lag sequential analysis and the Z_sum_ statistic (Cochran, 1954). This statistic provides a representative value for a series of independent values (adjusted residuals at different prospective or retrospective negative lags) to produce prospective and retrospective Z_sum_ values. Sackett (1980) recommended using the same number of prospective and retrospective lags. Based on experience to date (Sackett, 1987; Anguera and Losada, 1999), we suggest analyzing at least five prospective lags and five retrospective lags (−5 to +5).

The results of the computation determine the quadrant in which the different vectors are located and indicate their respective lengths and angles (Sackett, 1980). Vectors provide information on the nature of the relationship (prospective/retrospective activation/inhibition) between a focal behavior, which is equivalent to a given behavior in lag sequential analysis, and other categories of interest, known as conditional behaviors. The concept of genuine retrospectivity (Anguera, 1997) was introduced at a later stage to improve the classic concept of retrospectivity. The genuine retrospective approach considers negative lags from a backwards rather than a forwards perspective, i.e., it looks at what happened from lag 0 back to lag −5 rather than from lag −5 to lag 0.

Adjusted residuals, *Z*-values, and vector length and angles can all be computed in the open-access software program HOISAN (v. 1.6.3.3) (Hernández-Mendo et al., 2012), which also includes a feature to produce the results in graph form.

The meaning of the vectors (see below) varies according to the quadrant in which they are located, and the position of a vector in one quadrant or another is determined by the combination of positive or negative signs on the prospective and retrospective Z_sum_ values. In quadrant I (+ +), the focal and conditional behaviors activate each other; in quadrant II (− +), the focal behavior inhibits and is activated by the conditional behavior; in quadrant III (− −), the focal and conditional behaviors inhibit each other; and in quadrant IV (+ −), the focal behavior activates and is inhibited by the conditional behavior. The length of the vectors indicates the strength (statistical significance) of the association between the focal and conditional behaviors.

To illustrate briefly how the technique works, we used the data from Table 3a to produce a vector map showing the relationships between A, the focal behavior (in our example, expressions of sorrow or sadness), and categories B (expressions of self-perceived improvement), C (expressions of self-perceived worsening), and D (expressions of joy at having overcome the problem), the conditional behaviors. Table 7 shows the values for the adjusted residuals and corresponding Z_sum_ values, while Table 8 shows the length and angle of the vectors for each of the conditional behaviors. The corresponding vectors are shown in Figure 4.

**Table 7:** Adjusted residuals and corresponding *Z*-values from the polar coordinate analysis with A as the focal behavior or category and B, C, and D as the conditional behaviors.

**Lag**	**Categories**			
	**A**	**B**	**C**	**D**
**ADJUSTED RESIDUALS**				
−5	1.94	−0.187	−0.996	−1.236
−4	−1.936	0.826	2.148	−0.413
−3	1.253	−1.557	−1.019	0.934
−2	−0.125	2.49	−1.112	−1.689
−1	−2.121	−1.861	2.121	2.605
0	4.359	−1.77	−1.282	−1.77
+1	−2.121	3.207	0	−0.744
+2	−0.125	−1.689	−0.078	1.9
+3	1.253	0.596	−0.165	−1.789
+4	−1.936	0.826	0.537	0.826
+5	1.94	−1.764	−1.139	0.706
**Z-VALUES**				
−5	1.94	−0.187	−0.966	−1.236
−4	−1.936	0.826	2.148	−0.413
−3	1.253	−1.557	−1.019	0.934
−2	−0.125	−2.49	−1.112	−1.689
−1	−2.121	−1.861	2.121	2.605
+1	−2.12	3.207	0	−0.74
+2	−0.13	−1.69	−0.08	1.9
+3	1.253	0.596	−0.165	−1.789
+4	−1.836	0.826	0.537	0.826
+5	1.94	−1.764	−1.139	0.706

*The analysis was performed in HOISAN*.

**Table 8:** Polar coordinate analysis results showing the length and angle of the different vectors, the quadrant in which each vector is located, and the Z_sum_ values (Cochran, 1954) from the prospective and retrospective perspectives.

**Catergory**	**Quadrant**	**Prospective**	**Retrospective**	**Length**	**Angle**
SC_A	III	−0.44	−0.44	0.63	225
SC_B	IV	0.53	−0.13	0.54	346.19
SC_C	II	−0.38	0.52	0.65	125.79
SC_D	I	0.4	0.09	0.41	12.6

*In the presented situation, A is the focal behavior, so the results show how expressions of sorrow or sadness activate expressions of self-perceived improvement (Quadrant IV) or joy at having overcome the problem (Quadrant I). The focal behavior is not self-generating (Quadrant III). Additionally, expressions of sorrow or sadness do not generate self-perceived worsening (Quadrant II), although self-perceived worsening does generate the focal behavior*.

The strongest association detected for the focal behavior A (apart from with itself) was with B (in quadrant IV, with a vector length of 0.54), followed by D (quadrant I, with a vector length of 0.41). Although A and C have the longest vector (0.65), the fact that C is located in quadrant II (because its angle is 125.79°) means that A inhibits rather than activates C. C does not appear because its excitatory activity was insignificant.

Readers can find numerous examples of the application of polar coordinate analysis in a wide range of fields in direct observation (e.g., Gorospe and Anguera, 2000; Herrero Nivela, 2000; Anguera et al., 2003; Castañer et al., 2016, 2017; López et al., 2016; Aragón et al., 2017; Morillo et al., 2017; Santoyo et al., 2017; Suárez et al., 2018), and more recently indirect observation (e.g. Arias-Pujol and Anguera, 2017).

#### T-pattern detection

T-pattern detection was proposed and developed by Magnusson (1996, 2000, 2005, 2016). It involves the use of an algorithm that calculates the temporal distances between behaviors and analyzes the extent to which the critical interval remains invariant relative to the null hypothesis that each behavior is independently and randomly distributed over time. It needs data, in the form of code matrices, for which the duration of each co-occurrence has been recorded. Microanalyses of data are also possible and very useful (Anguera, 2005). The software program, Theme (v. 6 Edu), features different settings that can be modified to obtain complementary results that, analyzed together, can provide a greater understanding of interactive transitions over time. Theme is an open-access software program that provides all the necessary features for analyzing data and presenting the results graphically as dendrograms or tree diagrams.

As with lag sequential and polar coordinate analysis, we have also used the data from Table 3a to illustrate the use of T-pattern detection. It should be noted that the method applied is rather unconventional, as the temporal distance parameter was set at 1 in all cases.

**Figure 5:**
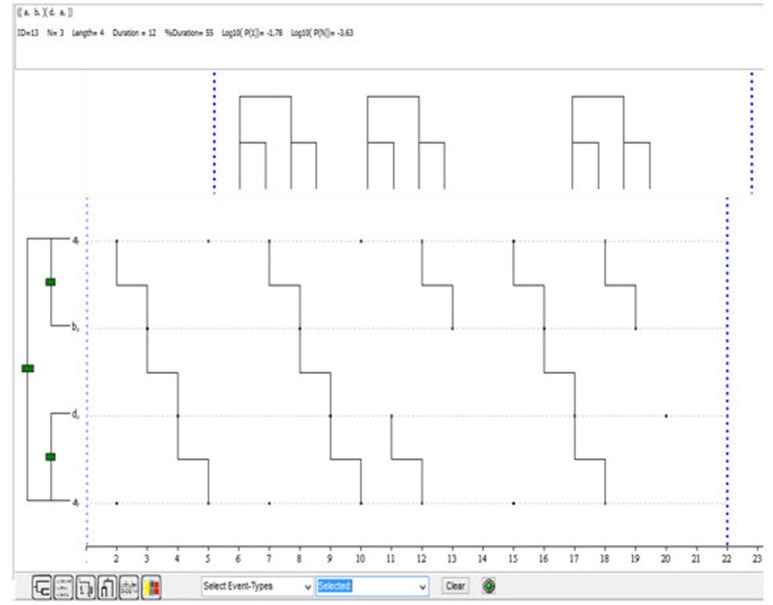
First of the 13 T-patterns detected in the data from Table 3a (*p* < 0.05).

Figure 5 shows the first of the 13 T-patterns obtained (*p* < 0.05). Note that despite the small size of the dataset, Theme detected a primary relationship between A and B (between expressions of sorrow or sadness and expressions of self-perceived improvement) and A and D (between expressions of sorrow or sadness and expressions of joy at having overcome the problem), as shown graphically in Figure 5.

Examples of the application of T-pattern detection can be found in studies by Castañer et al. (2013), Diana et al. (2017), Lapresa et al. (2013), and Sarmento et al. (2015) in direct observation and by Blanchet et al. (2005) and Baraud et al. (2016) in indirect observation.

#### Complementary use of techniques

Although the specifics of lag sequential analysis, polar coordinate analysis, and T-pattern detection differ, all three techniques serve to analyze and increase understanding of the internal structure of verbal or textual material derived from indirect observation. In addition, they can be applied to the same data to provide complementary insights and unveil invisible structures hidden within data. Their relevance is even greater in indirect observation studies where data have traditionally been analyzed from a purely qualitative perspective.

The convergence of results from three different quantitative approaches is a cause for celebration in a field such as indirect observation, where studies to date have largely relied on frequency counts or on qualitative approaches, which of course have their merits but are prone to considerable subjectivity bias.

There is growing interest in combining these techniques to gain a greater understanding of behavioral patterns that remain hidden to the naked eye. Two recent examples can be found in the studies of Santoyo et al. (2017) and Tarragó et al. (2017).

## Adapted methodological procedure for conducting an indirect observation study based on text liquefaction

We have presented a structured procedure detailing the successive stages of the method we propose for studying verbal behavior and/or textual material in an indirect observation study (Table 9). Our aim was not to offer a general approach to systematic observation from the perspective of indirect observation, as guidelines already exist for the reporting of systematic studies within observational methodology (Portell et al., 2015a). Our aim rather was to introduce the reader to the key concepts of indirect observation studies and provide step-by-step guidance on how to perform such a study. The procedure we propose is summarized in Table 9 and has already been applied in studies from different fields (Vaimberg, 2010; García-Fariña et al., 2016; Arias-Pujol and Anguera, 2017).

**Table 9:** Procedure for conducting an indirect observation study based on liquefying a text.

**Methodological action**	**Description**
**Guidelines for Indirect Observation (Liquefying a Text)**	
Define the research question	Focus the question on aspects that can be clearly delimited.
Delimit the source(s) of verbal or textual information using clearly specified criteria (setting, participants, situations, etc.)	Take all necessary decisions about the sources of data, such as type, volume, time frame, geographic location (if relevant) vs. online, etc.
Specify the study dimensions	After a detailed analysis of the theoretical framework, decide on the dimensions (or facets) of the research question.
Establish the text segmentation criteria	If the study is multidimensional, choose the primary dimension and define the segmentation criteria accordingly. This step will influence the segmentation of all the other dimensions, which will be considered secondary for this purpose.
Build an *ad hoc* observation instrument	Consider the number of dimensions, the existence or not of a theoretical framework, and the temporal nature of the subject of study (process vs. atemporal situation). Validate the coding process.
Code the data and create code matrices	Apply the codes from the observation instrument to the data to transform or ‘liquefy’ the primary material (verbal behavior or textual material) into matrices of codes suited for quantitative analysis.
Computerize the codes	Depending on the features of the software programs available, convert the dataset into a computerized format.
Merge/divide the code matrices in accordance with specific research questions	These data block management operations must be highly flexible as it may sometimes be necessary to jointly analyze several code matrices or to analyze parts of the same matrix separately.
Apply rigorous data quality controls	Rigorous data controls prior to the analysis of the data are essential for preventing possible biases from skewing the results. Such controls are necessary as the datasets are prone to subjectivity bias. Each set of textual units should be coded at least three times (by the same observer or by different observers).
Analyze the data quantitatively using a suitable technique or techniques	Once the quality of the dataset has been confirmed, the code matrices can be analyzed quantitatively to reveal underlying structures in the form of significant associations between codes. Choose the most appropriate technique depending on the aim of the analysis. Use lag sequential analysis to discover behavioral patterns that occur more often than would be expected by chance. Use polar coordinate analysis to obtain a vector map showing the prospective and retrospective activating or inhibitory relationships between a focal behavior and other behaviors of interest. Use T-pattern detection to uncover temporal relationships between categories based on the time distance between successive occurrences of each code.
Analyze convergent or complementary results if various techniques have been used	Compare and analyze similarities detected using different techniques and explore possible explanations for divergent results.
Evaluate the strengths and weaknesses of the study	The detection of strengths will allow you to argue the objectivity, rigor, and robustness of the study. Consider potential weaknesses by critically appraising the methodology, studying the literature, and evaluating the consistency of the theoretical framework.
Interpret your results	Interpret your results by analyzing them in the light of your research question(s) and considering similarities and differences reported by related studies.
Conduct a thorough and up-to-date literature review (although this is mentioned as the last step, relevant literature should be investigated and read throughout the study)	Conduct an exhaustive preliminary literature review and then apply rigorous filters as appropriate.

## Conclusions and limitations

Within the broad framework of mixed methods, we have presented indirect observation as a structured method consisting of different steps designed to guarantee scientific rigor. The method consists of the quantitization of qualitative data derived from verbal or textual material to produce code matrices which, following appropriate organization and rigorous quality control procedures, can be analyzed using robust, rigorous, and objective techniques. In a sense, we liquefy the text into a form suitable for quantitative analysis.

Although the materials that support direct and indirect observation are different, the methodological proposal described in this paper shows that both forms of observation share a systematic procedure in which adequately trained observers apply a robust, reliable purpose-designed observation instrument to produce quantitative indicators of the many processes underlying everyday behavior. The main strengths of our approach are that it enables the merging of data from different sources and offers the possibility of taking advantage of the continuous advances in information and communication technologies to study aspects of biopsychosocial behavior in everyday contexts. There are two main limitations. On the one hand, the dimensions in an indirect observation study depend largely on a theoretical framework and a conceptual framework, and these may be lacking. On the other hand, observation instruments comprising category systems, either alone or combined with a field format, also require a theoretical framework. However, the proposed approach has the advantage of allowing all data obtained from narratives to be included in the study, even those which do not fit with the theoretical framework or are contradictory. In fact, the validation of the coding process entails, among other things, checking that no new information has been added, that no information has been eliminated, and that the meaning of the information has not been altered. In this way, there is no omission of information that could lead to bias. This information can be included using bottom-up or top-down processes (Anguera, 1991; Anguera et al., 2007), in other words, the narratives are categorized on the basis of the chosen theoretical framework (top-down) and the theoretical framework is adapted on the basis of the narratives given (bottom-up). An exclusively quantitative study would entail the loss of sensitive and relevant information about the spontaneous behavior, as it would require excluding all variables not envisaged in the chosen theoretical framework. Hence our insistence on the enormous potential of mixed methods research, which suitably integrates both qualitative and quantitative elements.

This work presented a novel approach, based on sequence of occurrence, for transforming qualitative data into quantitative data that can be analyzed using robust quantitative techniques. Additionally, it is important to note that it is possible, at any time during the analysis, to return from the quantitative data to the narrative data. As a result, this approach presents advantages of both qualitative and quantitative methods, at the same time it covers weaknesses of both methods.

## Author contributions

All authors listed have made a substantial, direct and intellectual contribution to the work, and approved it for publication.

### Conflict of interest statement

The authors declare that the research was conducted in the absence of any commercial or financial relationships that could be construed as a potential conflict of interest.
